# Isometric hip strength impairments in patients with hip dysplasia are improved but not normalized 1 year after periacetabular osteotomy: a cohort study of 82 patients

**DOI:** 10.1080/17453674.2020.1864911

**Published:** 2021-02-04

**Authors:** Julie Sandell Jacobsen, Stig Storgaard Jakobsen, Kjeld Søballe, Per Hölmich, Kristian Thorborg

**Affiliations:** a Research Centre for Health and Welfare Technology, Programme for Rehabilitation, VIA University College, Aarhus;; b Research Unit for General Practice in Aarhus, Aarhus;; c Department of Orthopaedic Surgery, Aarhus University Hospital, Aarhus;; d Department of Clinical Medicine, Aarhus University, Aarhus;; e Sports Orthopaedic Research Center-Copenhagen (SORC-C), Department of Orthopaedic Surgery, Copenhagen University Hospital, Hvidovre;; f Physical Medicine and Rehabilitation Research-Copenhagen (PMR-C), Department of Physical and Occupational Therapy, Copenhagen University Hospital, Hvidovre, Denmark

## Abstract

Background and purpose — In patients with hip dysplasia, knowledge of hip muscle strength after periacetabular osteotomy is lacking. We investigated isometric hip muscle strength in patients with hip dysplasia, before and 1 year after periacetabular osteotomy, and compared this with healthy volunteers. Furthermore, we investigated whether pre- to post-surgical changes in self-reported pain and sporting function were associated with changes in isometric hip muscle strength.

Patients and methods — Isometric hip muscle strength was assessed twice in 82 patients (11 men) with a mean age of 30 (SD 9) years, before and 1 year after surgery, and once in 50 healthy volunteers. Isometric hip muscle strength was assessed with a hand-held dynamometer. Copenhagen Hip and Groin Outcome Score was used to measure self-reported outcome.

Results — Despite 1-year improvements in isometric hip flexion (0.1 Nm/kg; 95% CI 0.06–0.2) and abduction (0.1 Nm/kg; CI 0.02–0.2), the patients’ muscle strength was 13–34% lower than the strength of the healthy volunteers both pre- and post-surgery (p < 0.01). Moreover, changes in self-reported pain were associated with changes in hip flexion (13 points per Nm/kg; CI 1–26) and abduction (14 points per Nm/kg; CI 3–25), while changes in self-reported sporting function were associated with changes in hip extension (9 points per Nm/kg; CI 1–18).

Interpretation — Isometric hip muscle strength is impaired in symptomatic dysplastic hips measured before periacetabular osteotomy. 1 year after surgery, isometric hip flexion and abduction strength had improved but muscle strength did not reach that of healthy volunteers.

Hip dysplasia, with a prevalence of 5%, can be asymptomatic but in some cases, pain presents in early adulthood, most commonly among young women aged 24–35 years ((Jacobsen and Sonne-Holm 2005, Nunley et al. 2011, Jacobsen et al. 2018b) These patients are at risk of developing osteoarthritis at an early age (Murphy et al. 1995, Wyles et al. 2017).

The periacetabular osteotomy (PAO) is the preferred treatment for symptomatic patients with hip dysplasia in Western Europe, North America, and Australia (Lerch et al. 2017). Numerous studies have shown large improvements in self-reported pain, sporting function, and quality of life after PAO (Clohisy et al. 2017, Wasko et al. 2019). Nevertheless, little is known about what patients can expect with regard to physical capacity. In a study of 41 patients undergoing PAO (Mechlenburg et al. 2018), patients improved their leg extension power at 1-year follow-up to no differences between the operated and contralateral leg. Contrary to these findings, no changes in isometric hip flexor and abductor strength were found 2 years after PAO in a study of 22 patients, and their hip muscle strength was impaired compared to the strength of 29 healthy volunteers (Sucato et al. 2015). It is noteworthy, in both studies, that physical capacity was investigated in small populations and with conflicting results. Physical capacity should better be investigated prospectively in larger populations to gain knowledge on what patients can expect 1 year after PAO.

We investigated isometric hip muscle strength in patients with hip dysplasia, before and 1 year after periacetabular osteotomy, and compared this with healthy volunteers. Furthermore, we investigated whether pre- to post-surgical changes in self-reported pain and sporting function were associated with changes in isometric hip muscle strength.

## Patients and methods

### Study design

This is a prospective cohort study with 1-year follow-up, comparing isometric hip muscle strength between patients with hip dysplasia and healthy volunteers. The study is part of a prospective investigation on the same patient population, reporting pain and ultrasonographic abnormalities in hip muscles and tendons and physical activity level before and after PAO (Jacobsen et al. 2018a, 2018b, 2018c, 2019).

### Setting

From May 2014 to August 2015, we prospectively recruited patients with bilateral or unilateral hip dysplasia from the Department of Orthopedics at Aarhus University Hospital in Denmark (Jacobsen et al. 2018b). Later, from February 2019 to May 2019, we recruited healthy volunteers through personal networks, through invitations in social media, and from local private companies and public institutions (e.g., university hospitals, universities, and university colleges). We measured participant characteristics and outcomes at a clinical examination scheduled twice in the patients, before and 1 year after PAO, and once in the healthy volunteers.

### Participants

Patients with hip dysplasia were eligible for inclusion in this study if they had groin pain for at least 3 months, if Wiberg’s center–edge (CE) angle was < 25 degrees and if they were scheduled to undergo PAO. Patients with known comorbidities or a history of previous trauma or surgical interventions affecting the hip were excluded (Jacobsen et al. 2018b). The healthy volunteers were eligible if they were of same age and sex as the patients (not matched 1:1). Specifically, they were included if they were 18–49 years of age (i.e., equal to the patients), had a BMI of 18–26 (i.e., equal to the patients), and had no pain in back, hip/groin, knee, or ankle joint. We excluded healthy volunteers diagnosed with known hip dysplasia, healthy volunteers who performed sport at elite level and healthy volunteers having > 1 of the above listed exclusion criteria of the patients.

### Participant characteristics

Characteristics of the participants including age, sex, preferred sports, time in preferred sports, and time in general physical activity were recorded through standardized questions. Back pain intensity was measured with the Oswestry Disability Index (Fairbank et al. 1980). Pain at rest was measured on a numerical pain rating scale (NPRS) from no pain to unbearable pain (0–10). Hip-related pain was assessed with the Flexion/Adduction/Internal Rotation (FADIR) test and the Flexion/Abduction/External Rotation test (FABER) test (Troelsen et al. 2009). Occurrence of internal snapping hip was assessed with a standardized clinical test (i.e., moving from FABER position into extension, adduction, and internal rotation) (Tibor and Sekiya 2008), while back pain was assessed with posterior to anterior spring testing over the lumbar spinous processes (Schneider et al. 2008). SSJ measured the CE angle (Wiberg 1939), the Tönnis acetabular index angle, and the Tönnis osteoarthritis grade using standardized standing anteroposterior radiographs, while height and weight were measured and used to calculate BMI.

### Periacetabular osteotomy

After baseline examination, all patients underwent the minimally invasive approach for PAO performed by SSJ and KS (Jacobsen et al. 2019). The surgical procedure has been described previously (Troelsen et al. 2008). However, in short, the acetabulum was reoriented through 3 separate osteotomies aiming to improve the coverage of the femoral head. Post-surgery, the patients received in-hospital standardized rehabilitation including active range-of-motion exercises in lying and standing, and stair and gait training with crutches. The patients were discharged after approximately 2 days. For the first 6–8 weeks, the patients were allowed only partial weight-bearing with a maximum load of 30 kg. After discharge, the patients followed individualized physiotherapy-led rehabilitation for 2–4 months, including 2 weekly training sessions in groups.

### Outcomes

#### Muscle strength

GHM and JSJ assessed isometric hip muscle strength in the index limb with a handheld dynamometer (PowerTrack II Commander, JTECH Medical, Salt Lake City, UT, USA). The examiners used a standardized reliable dynamometer technique previously published by Thorborg et al. (2010). Isometric hip muscle strength was assessed with a make test in sitting position for flexion, in supine position for abduction and adduction, and in prone position for extension. The order of the individual strength assessments was randomized in order to avoid systematic bias.

The participants were instructed to stabilize themselves by holding on to the side of the examination table. They were instructed to practice 2 sub-maximal contractions, the 1st into the examiner’s hand and the 2nd into the dynamometer. During assessment, the participants exerted a 5-second maximum voluntary contraction against the dynamometer. We normalized all strength values to moment arms and weight and reported strength values in Nm/kg bodyweight. After each muscle strength assessment (i.e., hip flexion, extension, abduction, and adduction) patients verbally rated pain during maximum muscle contraction on a NPRS. The inter-rater reliability of the muscle strength assessments was investigated by Jacobsen et al. (2018b). The intraclass correlation coefficient was > 0.70 for all muscle strength assessments and the standard error of measurement ranged between 9.5% and 14%.

#### Self-reported outcome

Self-reported outcome was measured with the Copenhagen Hip and Groin Outcome Score (HAGOS) (Thorborg et al. 2011a). HAGOS is designed to measure self-reported outcome in young to middle-aged patients with hip and/or groin pain and measures self-reported outcome from 0 to 100 points on 6 separate subscales. These subscales are pain, symptoms, physical function in daily living (ADL), physical function in sports and recreation (sporting function), participation in physical activity (participation), and quality of life. HAGOS is a reliable, valid, and responsive outcome measure, associated with correlation coefficients of 0.2–0.7 across sub-items when correlated to relevant constructs. The measurement error ranges from 1 to 5 points across sub-items at the group level (Thorborg et al. 2011a, Kemp et al. 2013, Thomeé et al. 2014). Besides HAGOS symptoms and ADL, the sub-items have a high responsiveness, reported as effect sizes of 1.1–1.9 (Thorborg et al. 2011a, Thomeé et al. 2014).

### Sample size considerations

As mentioned earlier, this study is part of a prospective investigation on the same patient population (Jacobsen et al. 2018a, 2018b, 2018c, 2019). Thus, we did not perform an ordinary sample size calculation for this study as the numbers of patients were fixed when planning this analysis.

### Statistics

Normally distributed continuous data were reported as means (SD), otherwise reported as medians with either ranges or interquartile ranges (IQR). Categorical data were reported as numbers. Differences in hip muscle strength (i.e., flexion, extension, abduction, and adduction) and self-reported outcome (6 HAGOS subscales) between groups and changes from baseline to 1-year follow-up in the patients were analyzed with a mixed-effect model with patients as random factors, and time and group (i.e., patients or healthy volunteers) as fixed factors. Model assumptions were based on inspection of plots of standardized residuals versus fitted values and quantile–quantile (QQ) plots of the standardized residuals. Differences were reported as means with 95% confidence intervals (CI). Simple linear regression analyses were performed with hip strength of each muscle group as independent variables (i.e., flexion, extension, abduction, and adduction), and the HAGOS subscales covering pain and sporting function as the dependent variables. Crude β coefficients were reported with CI, and the model assumptions of the linear regression analyses were based on inspection of scatter plots, QQ plots of the standardized residuals and plots of standardized residuals versus fitted values. The level of significance was 0.05 and the STATA 14.2 (StataCorp, College Station, TX, USA) software package was used for the data analysis.

### Ethics, registration, funding, and potential conflicts of interest

Ethical approval was obtained from the Central Denmark Region Committee on Biomedical Research Ethics (patients: 5/2014 and volunteers: 252/2018). The Danish Data Protection Agency authorized patient data handling (1-16-02-47-14), and the study protocol was registered at ClinicalTrials.gov (20140401PAO). The study was performed in accordance with the Code of Ethics of the World Medical Association and the Danish Code of Conduct for Research Integrity. All patients and healthy volunteers gave informed consent to participate. The authors declare that they have no potential conflicts of interest. This study was kindly supported by the Danish Rheumatism Association (Grant number A3280), the Aase and Ejnar Danielsen Fund (Grant number 10-000761/LPJ), and the Fund of Family Kjaersgaard, Sunds (Grant number 6041401).

## Results

**Figure 1.**
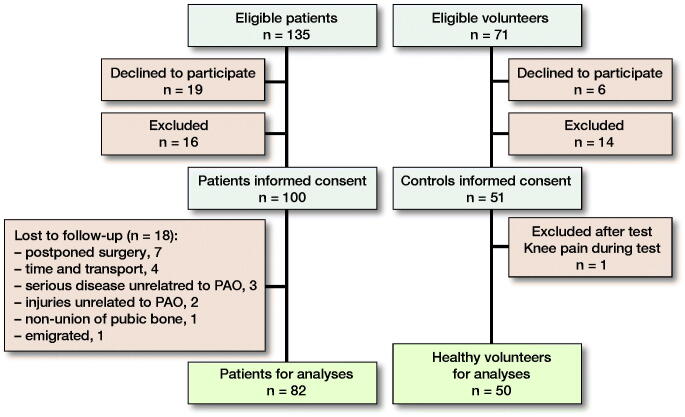
Flow of patients and healthy volunteers.: PAO = periacetabular osteotomy.

135 patients were assessed for eligibility (Figure 1). 19 patients declined to participate and 16 patients were excluded (Jacobsen et al. 2018b). The patients who declined to participate were, on average, somewhat younger (25 years; SD 9) compared with study participants completing 1-year follow-up but included a similar percentage of men (10%). Finally, 100 consecutive patients fulfilled the in- and exclusion criteria and gave informed consent to participate. Similarly, of 71 eligible healthy volunteers, 50 volunteers gave informed consent to participate.

At 1-year follow-up, 18 patients were lost to follow-up, givingn 82 patients for the follow-up analyses. The patients lost to follow-up were comparable to patients completing 1-year follow-up regarding age, BMI, physical activity, NRPS, CE, and AI angle (data not shown). The median follow-up time was 1.0 year (0.8–1.6). The patients and healthy volunteers were comparable in age, sex, and BMI (Table 1). However, the patients were less physically active and reported more back pain compared with the healthy volunteers.

**Table 1.** Baseline characteristics of patients with hip dysplasia and healthy volunteers. Values are count unless otherwise specified

Characteristics	Patients with hip dysplasia (n = 100)	Healthy volunteers (n = 50)
Mean age (SD)	30 (9)	31 (9)
Mean BMI (SD)	23 (3)	23 (3)
Men	17	8
Preferred sports, h/week (IQR)	0 (2)	4 (10)
Preferred sports		
Fitness	28	17
Running	19	10
Team sports	13	12
Gymnastics	7	3
Horseback riding	6	0
Racket sports	2	2
Swimming	2	0
Dancing	2	0
Other ^a^	18	6
No preferred sports	3	0
General physical activity h/week		
< 2.5 h/week	13	3
2.5 to < 5 h/week	19	7
5 to < 10 h/week	42	30
≥ 10 h/week	26	10
Back pain intensity		
No	31	41
Very mild	23	7
Moderate	26	2
Fairly severe	14	0
Very severe	5	0
Worst imaginable	1	0
Posterior to anterior spring testing (SP)		
Hip pain	12	0
Back pain	35	6
No pain	53	44
Positive FADIR test	83	3
Positive FABER test	74	2
Positive internal snapping hip test	30	0
Bilateral affection	89	–
NRS pain (range)	3 (2–5)	–
Centre-edge angle (°; SD)	17 (5)	–
Tцnnis acetabular index angle (°; SD)	14 (5)	–
Osteoarthritis grade 0/1	97/3	–

a Covers different combat and self-defense sports, bicycling and hiking.

IQR = interquartile range,

SP = spinous processes,

FADIR = flexion/adduction/internal rotation,

FABER = flexion/abduction/external rotation,

NRS = numerical rating scale.

### Muscle strength

**Figure 2.**
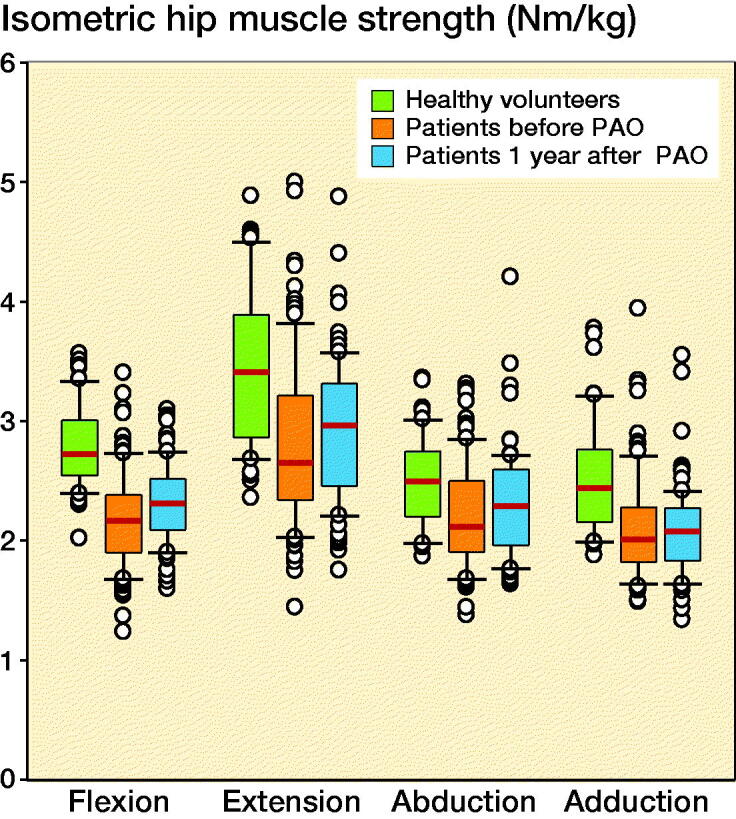
Median isometric hip muscle strength in patients with hip dysplasia and in healthy volunteers in Nm/kg; box represents 25th and 75th percentiles and error bars represent 10th and 90th percentiles. PAO = periacetabular osteotomy.

The patients improved their hip muscle strength statistically significantly from before to 1 year after PAO in hip flexion and abduction, whereas muscle strength hip extension and adduction were unchanged (Table 2). Moreover, both pre- and 1-year post-surgery, patients had 13–34% lower hip muscle strength compared with the healthy volunteers, covering all muscle groups (Figure 2, Table 3).

**Table 2.** Mean changes in isometric hip muscle strength in Nm/kg

Hip strength	Volunteers		Before PAO		1 year after PAO		HD change (95% CI)	HD change	
	n	mean (SD)	n	mean (SD)	n	mean (SD)		(%)	p-value
Flexion	50	1.8 (0.34)	100	1.2 (0.40) ^a^	80	1.3 (0.32) ^a^	0.13 (0.06 to 0.20)	11	< 0.001
Extension	50	2.5 (0.63)	97	1.8 (0.68) ^a^	80	1.9 (0.59) ^a^	0.09 (–0.05 to 0.23)	5	0.2
Abduction	50	1.5 (0.37)	99	1.2 (0.43) ^a^	81	1.3 (0.44) ^a^	0.10 (0.02 to 0.18)	8	0.02
Adduction	50	1.5 (0.46)	98	1.1 (0.44) ^a^	80	1.1 (0.37) ^a^	–0.03 (–0.11 to 0.06)	–3	0.5

a Statistically significantly lower than the values of the healthy volunteers (p < 0.01).

PAO = periacetabular osteotomy.

**Table 3.** Mean differences in muscle strength in Nm/kg between 100 patients and 50 volunteers

Hip strength	Before PAO			1 year after PAO		
	Mean diff. (95% CI)	% diff.	p-value	Mean diff. (95% CI)	% diff.	p-value
Flexion	0.60 (0.48–0.73)	34	< 0.001	0.47 (0.34–0.60)	27	< 0.001
Extension	0.66 (0.44–0.87)	27	< 0.001	0.56 (0.34–0.79)	23	< 0.001
Abduction	0.30 (0.16–0.44)	20	< 0.001	0.20 (0.05–0.35)	13	0.008
Adduction	0.41 (0.27–0.56)	27	< 0.001	0.44 (0.29–0.59)	29	< 0.001

PAO = periacetabular osteotomy.

The median pre-surgical pain intensity levels during each muscle strength assessment were 3 (0–10) in hip flexion, 2 (0–10) in hip extension, 4 (0–10) in hip abduction, and 2 (0–10) in hip adduction. The median post-surgical levels were 1 (0–8) in hip flexion, 0 (0–8) in hip extension, 1 (0–8) in hip abduction, and 1 (0–10) in hip adduction.

### Self-reported outcome

**Figure 3.**
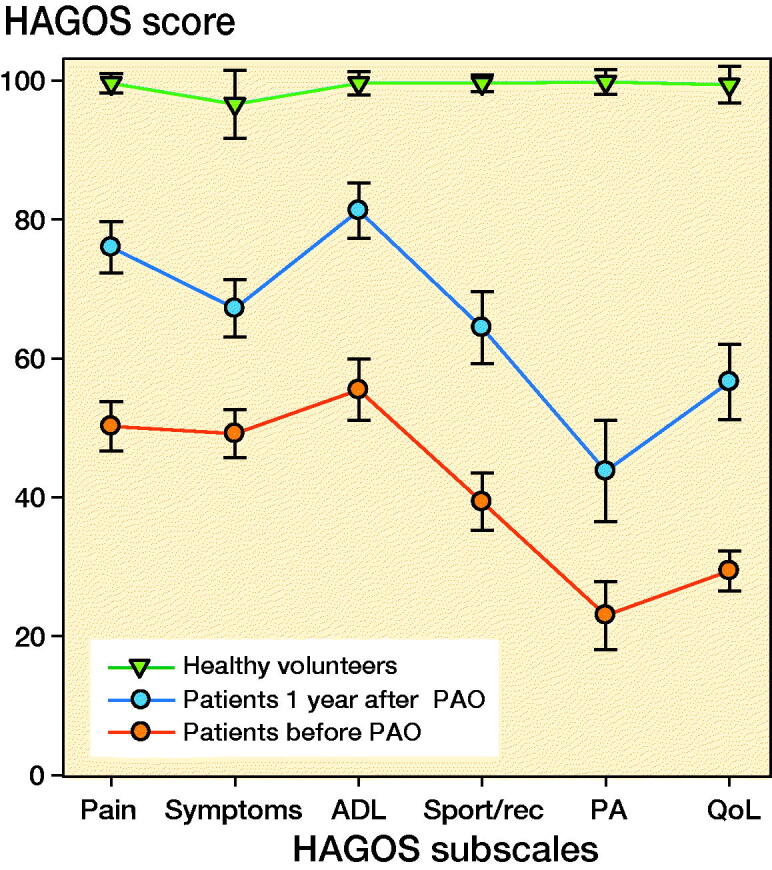
Profile of Copenhagen Hip and Groin Outcome Score (HAGOS) in patients and healthy volunteers in HAGOS points (0–100); error bars represent 95% confidence intervals. PAO = periacetabular osteotomy, ADL = physical function in daily living, Sport/rec = physical function in sports and recreation, PA = participation in physical activity, QoL = quality of life.

From before to 1 year after PAO, all HAGOS subscales improved statistically significantly. However, the patients reported lower self-reported outcome both pre- and 1-year post-surgery compared with the healthy volunteers (Figure 3).

### Associations between HAGOS and muscle strength

Changes in HAGOS pain and sporting function were positively associated with changes in hip muscle strength (Table 4), where an increase of 1 Nm/kg in hip flexion, extension, and abduction was associated with 9–14-point higher HAGOS score in pain and sporting function.

**Table 4.** Associations of change in HAGOS to change in muscle strength in HAGOS points/Nm/kg. Values are crude β coefficients with 95% confidence interval (CI) for associations between HAGOS and hip muscle strength measured before and 1 year after periacetabular osteotomy

Outcomes	Hip flexion (n = 80)		Hip extension (n = 78)		Hip abduction (n = 81)		Hip adduction (n = 79)	
	β (CI)	p-value	β (CI)	p-value	β (CI)	p-value	β (CI)	p-value
HAGOS pain	13.4 (0.5 to 26.3)	0.04	6.3 (–0.5 to 13.1)	0.07	14.0 (3.3 to 24.7)	0.01	7.6 (–3.3 to 18.5)	0.2
HAGOS sport	13.2 (-4.1 – 30.5)	0.1	9.3 (0.6 to 17.9)	0.04	11.6 (–2.9 to 26.1)	0.1	0.9 (–13.5 to 15.4)	0.9

HAGOS = Copenhagen Hip and Groin Outcome Score, sport = physical function in sport and recreation.

## Discussion

We found that patients with hip dysplasia improved their isometric hip muscle strength in hip flexion and abduction by 8–11%. Compared with healthy volunteers, the hip muscle strength of the patients was impaired both pre- and post-surgery, and the pre- to post-surgical changes in hip muscle strength were associated with self-reported outcome.

In a previous study, isometric hip muscle strength was investigated in 22 patients with hip dysplasia undergoing the abductor-sparing approach for PAO (Ezoe et al. 2006). Compared with our results, the pre-surgical hip muscle strength improved somewhat more (0.6–0.8 Nm/kg to 0.8–0.9 Nm/kg). Moreover, similar to our results, the pre-surgical isometric hip muscle strength was 25–46% lower in all muscle groups than the hip muscle strength of 24 healthy volunteers, and the 12-months muscle strength was 27–31% lower. In another study, isometric muscle strength was investigated pre-, 1-, and 2-year post-surgery in 22 hips undergoing PAO (Sucato et al. 2015). Contraty to our findings, the authors found no pre- to 2-year post-surgical changes in hip strength and only small insignificant changes from 1 to 2 years. Compared with the 29 healthy volunteers, the hip muscle strength was impaired both pre- and post-surgery. In summary, the results of the previous studies represent divergent findings. However, the previous studies do indicate that hip muscle strength is impaired in patients with hip dysplasia, and that small to moderate changes can be expected 1 year after PAO.

No previous studies have investigated associations between changes in self-reported outcome and hip muscle strength. However, correlations between HAGOS and performance-based outcomes have been investigated in 32 patients with hip dysplasia (Jacobsen et al. 2013). Statistically significant correlations were reported between self-reported pain and sporting function and kinetic gait variables (i.e., hip flexor joint moment measured with a motion-capture system in walking and running). Compared with our results, the correlations were somewhat smaller. Nevertheless, we found that an increase of 1 Nm/kg in hip muscle strength was associated with 9–14 higher HAGOS points, indicating clinically relevant associations. However, previous studies have reported hip muscle strength improvements of only 9–14% after 8 weeks of progressive resistance training (Mortensen et al. 2018), 3 months of hip abductor strengthening (Kuroda et al. 2013), and 6 weeks of task-specific training and progressive hip strengthening (Harris-Hayes et al. 2016). Furthermore, based on our results, a hip muscle strength improvement of 1 Nm/kg would correspond to an improvement of 56%, which is much higher than the higher limits of our and previous reported CIs on improved hip muscle strength, indicating that pre- to post-surgical changes in hip muscle strength were only to a lesser extent associated with changes in patient-reported outcome.

Pre- to post-surgical changes in hip muscle strength may occur due to multiple factors, covering biomechanical improvements, pain reduction, physical rehabilitation etc. 1st, in hip dysplasia, the hip joint is lateralized (Harris et al. 2017), implying that the hip abductors have to generate higher medially directed forces to produce normal movement (Skalshøi et al. 2015, Harris et al. 2017). Therefore, improving the biomechanical conditions with the PAO could possible also change the length tension relationships, which could positively change hip muscle strength. 2nd, pain intensity improved from pre-surgical levels of 2–4 to post-surgical levels of 0–1 using an NPRS, possibly improving the patients’ ability to perform maximum voluntary contractions as previously reported (Thorborg et al. 2011b). 3rd, the patients followed individualized physiotherapy-led rehabilitation for 2–4 months, possibly associated with improved hip muscle strength. On the other hand, self-reported time in preferred physical activities was much lower in the patients compared with the healthy volunteers (Table 1), and the patients were less physical active compared to the healthy volunteers. The lower self-reported physical activity level of the patients may explain why the patients had larger strength deficits compared with the healthy volunteers and why only small improvements in hip muscle strength were found 1-year post-surgery. However, despite biomechanical improvements, pain reduction, and physical rehabilitation, the patients had impaired hip muscle strength 1-year post-surgery, which most likely also compromises function in strenuous activities such as running, stair climbing, jumping, and standing on 1 leg (Graven-Nielsen and Arendt-Nielsen 2008). The direct pathway from impaired muscle strength to reduced ability to perform everyday activities has not yet been established. However, our results indicate an association between hip muscle strength and self-reported hip function in patients rehabilitating from PAO surgery. Moreover, previous studies have suggested that low muscle strength is associated with pathological hip joint biomechanics and reduced dynamic postural control, and that these two are associated with hip osteoarthritis and lower extremity injury (Murphy et al. 2016, Wilson et al. 2018). Therefore, our results may have an implication for future patients as surgeons have the possibility to inform patients about the level of hip muscle strength that can be expected 1 year after PAO and to inform patients of possible consequences. Moreover, the low hip muscle strength 1-year post-surgery implies that future intervention studies should focus on improving hip muscle strength after PAO. To our knowledge, no studies have investigated the effect of different physical rehabilitation programs for patients with hip dysplasia.

### Methodological considerations

No attempt was made to monitor the post-surgical physical rehabilitation; our aim was to investigate pre- and post-surgical hip muscle strength in a setting comparable to usual care. Therefore, the observed post-surgical muscle strength may have been impacted by surgical biomechanical improvement, improved pain, the physical rehabilitation, or some combination of these factors. Furthermore, differences in age and sex could potentially affect estimates of differences between patients and healthy volunteers, as differences in age and gender are associated with muscle strength. However, we managed to control this by recruiting healthy volunteers of the same age and sex as the patients. Hence, we considered it unnecessary to control for this in our analyses. Moreover, the younger age among the patients who declined to participate could not bias our findings as non-participation before baseline test affects only generalizability. Finally, hip muscle strength was not assessed later than 1 year after PAO, and we cannot rule out that the hip muscle strength may continue to improve further. However, Sucato et al. (2015) investigated hip muscle strength pre-, 1, and 2 years post-surgery and found no changes in hip muscle strength post-surgery compared with pre-surgery and only small insignificant differences from 1 to 2 years post-surgery, indicating that the hip muscle strength has plateaued already after 1 year. Of note is that, despite the younger age among the patients who declined to participate, the generalizability of our findings is considered high and a strength of this study as the rate of eligible patients was similar to the general flow of patients at our institution.

## Conclusion

Isometric hip muscle strength is impaired in patients with symptomatic dysplastic hips measured before PAO. 1 year after surgery, isometric hip flexion and abduction strength had improved but muscle strength did not reach that of healthy volunteers.
