# Isometric hip strength impairments in patients with hip dysplasia are improved but not normalized 1 year after periacetabular osteotomy: a cohort study of 82 patients

**DOI:** 10.1080/17453674.2021.1986292

**Published:** 2021-10-11

**Authors:** Julie Sandell Jacobsen, Stig Storgaard Jakobsen, Kjeld Søballe, Per Hölmich, Kristian Thorborg

**Affiliations:** a Research Centre for Health and Welfare Technology, Programme for Rehabilitation, VIA University College, Aarhus;;; b Research Unit for General Practice in Aarhus, Aarhus;;; c Department of Orthopaedic Surgery, Aarhus University Hospital, Aarhus;;; d Department of Clinical Medicine, Aarhus University, Aarhus;;; e Sports Orthopaedic Research Center-Copenhagen (SORC-C), Department of Orthopaedic Surgery, Copenhagen University Hospital, Hvidovre;;; f Physical Medicine and Rehabilitation Research-Copenhagen (PMR-C), Department of Physical and Occupational Therapy, Copenhagen University Hospital, Hvidovre, Denmark

In the published article Figure 2 had a typographical error in the y-axis values which went from 0 to 6 now corrected to 0 to 5.

**Figure 2.**
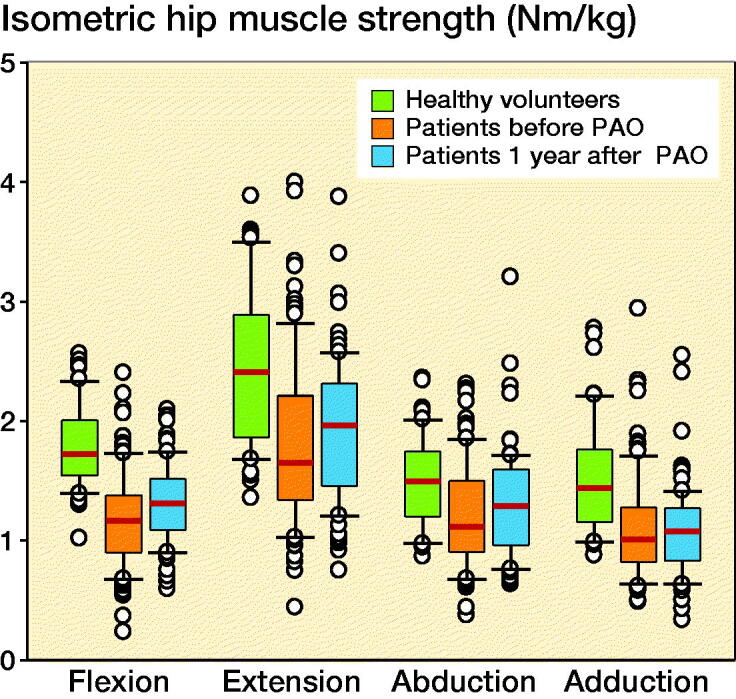
Median isometric hip muscle strength in patients with hip dysplasia and in healthy volunteers in Nm/kg; box represents 25th and 75th percentiles and error bars represent 10th and 90th percentiles. PAO = periacetabular osteotomy.

